# A randomized trial of oral gamma aminobutyric acid (GABA) or the combination of GABA with glutamic acid decarboxylase (GAD) on pancreatic islet endocrine function in children with newly diagnosed type 1 diabetes

**DOI:** 10.1038/s41467-022-35544-3

**Published:** 2022-12-24

**Authors:** Alexandra Martin, Gail J. Mick, Heather M. Choat, Alison A. Lunsford, Hubert M. Tse, Gerald G. McGwin, Kenneth L. McCormick

**Affiliations:** 1 Department of Pediatrics, Division of Pediatric Endocrinology, University of Alabama at Birmingham, Birmingham, AL USA; 2 Department of Microbiology, Comprehensive Diabetes Center, University of Alabama at Birmingham, Birmingham, AL USA; 3 Department of Epidemiology, School of Public Health, University of Alabama at Birmingham, Birmingham, AL USA

**Keywords:** Type 1 diabetes, Type 1 diabetes

## Abstract

Gamma aminobutyric acid(GABA) is synthesized by glutamate decarboxylase(GAD) in β-cells. Regarding Type 1 diabetes(T1D), animal/islet-cell studies found that GABA promotes insulin secretion, inhibits α-cell glucagon and dampens immune inflammation, while GAD immunization may also preserve β-cells. We evaluated the safety and efficacy of oral GABA alone, or combination GABA with GAD, on the preservation of residual insulin secretion in recent-onset T1D. Herein we report a single-center, double-blind, one-year, randomized trial in 97 children conducted March 2015 to June 2019(NCT02002130). Using a 2:1 treatment:placebo ratio, interventions included oral GABA twice-daily(*n* = 41), or oral GABA plus two-doses GAD-alum(*n* = 25), versus placebo(*n* = 31). The primary outcome, preservation of fasting/meal-stimulated c-peptide, was not attained. Of the secondary outcomes, the combination GABA/GAD reduced fasting and meal-stimulated serum glucagon, while the safety/tolerability of GABA was confirmed. There were no clinically significant differences in glycemic control or diabetes antibody titers. Given the low GABA dose for this pediatric trial, future investigations using higher-dose or long-acting GABA formulations, either alone or with GAD-alum, could be considered, although GABA alone or in combination with GAD-alum did nor preserve beta-cell function in this trial.

## Abstract (web summary)

Based on preclinical studies, gamma aminobutyric acid (GABA) and immunization for the enzyme that produces GABA glutamate decarboxylase (GAD) could be a potential therapy for type 1 diabetes. Here the authors report that in a placebo-controlled, double blind trial in children with new onset type 1 diabetes oral GABA plus GAD did not preserve beta-cell function measured as fasting/meal-stimulated c-peptide levels.

## Introduction

The pathogenesis of type 1 diabetes mellitus (T1D) entails autoimmune destruction of pancreatic beta cells^1–3^. Once hyperglycemia appears, more than 70% of islet beta cell mass has been eradicated^4^. Proliferation of surviving β-cells, pancreatic progenitor cells, plus transdifferentiation of alpha, acinar, ductal or hepatic cells, all have the potential to revitalize insulin production^5,6^.

Multiple immunological abnormalities have been reported in T1D patients including autoantibody production against the insulin molecule, the 65 kD isoform of glutamic acid decarboxylase (GAD65), various islet antigens, and the zinc transporter 8 (ZnT8) as well as decreased regulatory T cell (Treg) capacity to suppress T-cell mediated destruction of the islets of Langerhans^3^. To date, many studies attempting to ward off or reverse T1D have focused on immune suppression or modulation^7–11^, which may engender long-term side-effects. However, the recent antiCD3 antibody trials have shown a 3-year delay in clinical diagnosis of T1D^12,13^.

Animal and in vitro studies maintain that gamma aminobutyric acid (GABA) and glutamic acid decarboxylase (GAD) play fundamental metabolic roles in the pancreas and may be potential therapeutic targets in T1D. As for GAD65 antigen (GAD-alum) treatment per se in new onset T1D, an initial 2008 report of 70 patients was auspicious insofar as residual beta-cell function over 30 months was somewhat preserved. Yet a later, and more comprehensive, study with 334 patients failed to replicate this finding^14^. However, individual level analysis of these two studies and another^15^ found that study participants positive for HLA DR3-DQ2, but negative for HLA-DR4-DQ8, demonstrate enhanced beta cell preservation following GAD-alum monotherapy^16^.

GABA, a major inhibitory neurotransmitter, is abundant within pancreatic islet*s*^17,18^ and participates in paracrine regulation of β and α cells^19,20^. GAD, the enzyme that decarboxylates glutamate to form GABA, is a major autoantigen in T1D^3,21^. In vitro experiments found that isolated human islets treated with GABA receptor blockade have decreased insulin secretion at physiologic glucose concentrations^18^. Further, GABA-deficient islets did not show appropriate glucagon inhibition in response to increasing glucose concentrations in vitro^22^, suggesting that GABA is directly involved in the suppression of glucagon secretion in pancreatic alpha cells. GABA activates the Ca2+ - P13K/Akt growth and survival pathway and averts stress-induced apoptosis in islet cell lines treated in vivo with streptozotocin (STZ)^19^. In vivo, GABA delays diabetes onset in both the non-obese diabetic (NOD) and the STZ-treated mouse if given early in life^19^. And, if GABA treatment was initiated in NOD and STZ mice after diabetes had already commenced, normoglycemia ensued^19^. The mechanisms are not fully understood, but are proposed to involve tempering of the pancreatic autoimmune milieu and systemic inflammation.

Apart from demonstrating β-cell regeneration and glucagon suppression with GABA in two distinct diabetic mouse models, Soltani and colleagues described significant decreases in inflammatory cytokine expression^19^. Functional GABA receptors are present on T-cells and increases expression of splenic T regulatory cells, in turn potentially arresting or slowing T cell mediated beta cell destruction^19,23,24^. In vivo, GABA inhibits adoptive transfer of T1D following transplant of diabetogenic splenic T cells into a NOD/SCID mouse model. Individually, GABA and GAD-alum promote survival of transplanted beta cells in the NOD mouse, while combination therapy promoted synergistic and dose dependent beta cell survival^25^. To date, neither GABA alone, nor GABA-GAD in tandem, has been explored as therapeutic agents in study participants with T1D. Here we show, in this human trial of low-dose GABA, alone or as co-therapy GABA/GAD, that while the primary outcome, β-cell function, was not statistically proven, the combination GABA/GAD reduced fasting and meal-stimulated serum glucagon. Glycemic control, proinsulin and diabetes autoantibodies, all secondary outcomes, were similar between GABA/GAD and placebo. Moreover, the safety and tolerability of the treatments was established.

## Results

### Recruitment and tolerability of intervention

**Fig. 1:**
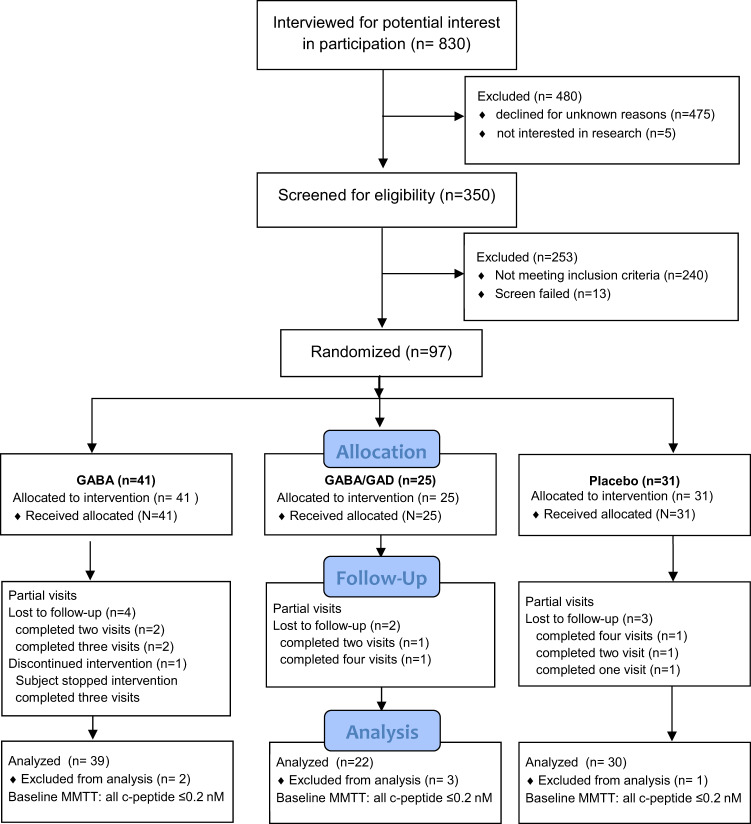
Consort profile. Participants, aged 4–18 years old, were screened at diagnosis with T1D and enrolled at our tertiary care university center at Children’s Hospital of Alabama in Birmingham, Alabama. Nine study participants were from out-of-state.

Between March 2015 and June 2018, 350 patients were screened and a total of 97 patients enrolled (Fig. 1). There were six unrelated serious adverse events recorded that required uneventful 1–2 day hospitalizations^26^.

### Patient characteristics

The baseline patient characteristics for each treatment group are summarized in Table 1. The age-stratified randomization was successful. The ethnic distribution was as follows: 90% Caucasian, 7% African American, 2% Hispanic and 1% Native American. All patients were diabetes antibody positive with most retaining positivity in three. There were no statistical differences regarding initial presentation, including, age, diabetes ketoacidosis, the number of positive diabetes antibodies, body mass index, HbA_1c_, fasting c-peptide or glucagon. All patients were enrolled by 5–6 weeks post diagnosis of T1D.

**Table 1:** Baseline participant characteristics

Parameter	GABA *n* = 39	GABA/GAD *n* = 22	Placebo *n* = 30	GABA vrs placebo	GABA/GAD vrs placebo
Age, years	11.2 ± 3.9	11.6 ± 3.2	11.1 ± 3.5	0.887	0.594
4–8 yrs (%)	31%	32%	30%	0.633	0.814
9–11 yrs(%)	44%	45%	53%		
14–18 yrs (%)	26%	23%	17%		
Sex Male %(*n*)	54%(21)	64%(14)	43%(13)	0.470	0.171
Female %(*n*)	46%(18)	36%(8)	57%(17)		
BMI (kg/m^2^)	19.6 ± 3.4	19.3 ± 3.4	19.0 ± 3.2	0.435	0.770
BMI percentile	66.9 ± 29.1	61.4 ± 26.5	60.3 ± 28.1	0.343	0.886
Ethnicity %(*n*)					
White	92.3% (36)	90.9% (20)	86.7% (26)	0.387	0.650
African American	5.1% (2)	9.1% (2)	6.7% (2)		
Hispanic	0	0	6.7% (2)		
Native American	2.6% (1)	0	0		
Days from diagnosis to baseline visit	25.3 ± 7.2	26.6 ± 6.3	25.8 ± 8.2	0.821	0.678
Diabetes ketoacidosis at diagnosis %(*n*)	23.0% (9)	22.7% (5)	36.6% (11)	0.287	0.368
Diabetes autoantibodies (% positive)^a^					
Anti-ICA 512	81%	87%	83%	0.814	1.000
Anti-Zinc Transporter-8	94%	74%	87%	0.407	0.282
Number of autoantibodies positive (% patients)					
1	3.9%	7.1%	8.3%		0.862
2	34.6%	28.6%	20.8%	0.503	
3	61.5%	64.3%	70.8%		
HbA1C %	11.0 ± 2.5	10.4 ± 2.2	11.1 ± 2.5	0.982	0.349
Total Daily Dose insulin (units/kg/day)	0.56 ± 0.21	0.47 ± 0.24	0.56 ± 0.21	0.984	0.167
C-peptide AUC at baseline (ng/ml/min)	1.85 ± 1.21	2.13 ± 1.16	1.87 ± 1.3	0.883	0.533
C-peptide fasting at baseline (ng/ml)	0.74 ± 0.55	0.78 ± 0.48	0.72 ± 0.60	0.884	0.630
Glucagon AUC at baseline (pg/ml/min)	78.05 ± 26.35	70.73 ± 24.51	77.90 ± 16.87	0.933	0.278
Glucagon fasting at baseline (pg/ml)	65.35 ± 16.10	61.11 ± 16.56	62.62 ± 13.62	0.906	0.770

Results are presented as mean ± SD unless otherwise specified. ^a^Anti GAD65 was a study inclusion criterion. Statistical comparisons were by two-tail analysis of variance or Chi square as indicated and as in Methods. GABA (gamma aminobutyric acid), GAD (GAD-alum).

### Effect of GABA alone and GABA/GAD in combination on c-peptide and glycemic control

**Fig. 2:**
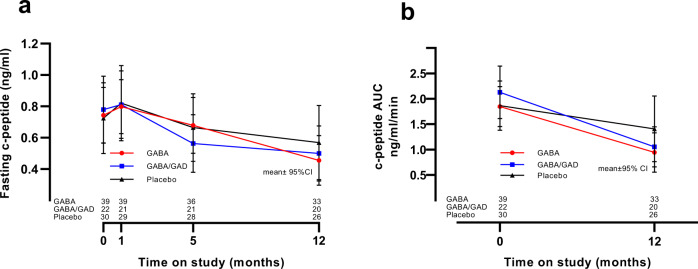
Fasting and AUC c-peptide in study groups over time. Fasting c-peptide (**a**) was measured in the three study groups (GABA-red, GABA/GAD-blue, and placebo-black) at baseline (Time = 0,prior any treatment) and at 1, 5 and 12-months thereafter. AUC c-peptide **(b)** was calculated over time in the three study groups. Results are given as mean ± 95% CI. No statistical differences were noted by two-sided analysis of covariance (complete statistical data is summarized in Supplementary Table 1). Source data are provided as a Source Data file.

**Fig. 3:**
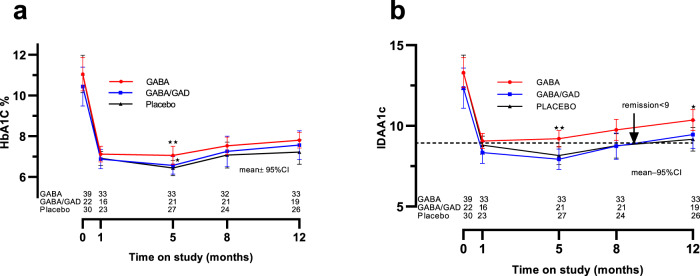
Glycemic control in study groups over time. Glycosylated Hemoglobin (HbA_1c_) (**a**) and insulin adjusted A1c (**IDAA1c**) (**b**) were measured in the three study groups. Results are shown as mean ±95% CI and statistical comparisons were by two-sided analysis of covariance. Regarding HbA1c (**a**) at 5-months GABA vrs. Placebo **p = 0.003 and GABA vrs GABA/GAD *p = 0.041. For IDAA1c (**b**) at 5-months, GABA vrs. Placebo **p = 0.007 and GABA vrs. GABA/GAD **p = 0.002. At 12-months, GABA vrs. Placebo *p = 0.020. Source data are provided as a Source Data file.

There was no statistical effect of oral GABA alone or combination GABA/GAD therapy on the primary outcome measure c-peptide, including both fasting and MMTT-stimulated area under the curve (Fig. 2a, b). The 90 min post MMTT c-peptide values for each study group are shown in Supplementary Fig. 1. As expected, there was a gradual diminution in c-peptide post diagnosis. A tabular summary of the statistical comparisons for the primary and secondary outcomes are presented in Supplementary Table 1. There was no statistical differences in HbA_1c_ outside of a small disparity in GABA versus placebo only at the 5-month visit, and none in GABA/GAD versus placebo at all study visits. To address this further, an analysis of area under the curve (AUC) glucose at baseline and 12-months as well as fasting glucose at baseline, 1 month, 5-months and 12-months showed no differences (Supplementary Fig. 9). Insulin dose-adjusted A1c (IDAA1c)^27^ was 12% increased in GABA compared to placebo at 5 and 12-months. By contrast, IDAA1c in GABA/GAD was not different from placebo at any time point (Fig. 3a, b). Importantly, applying the gold-standard reference used to establish IDAA1c, namely, a meal-stimulated c-peptide >300 pM^27^, did not reveal statistical differences between the groups (Supplementary Fig. 8a). Moreover, a sub-analysis of IDAA1c in those participants who transitioned from basal/bolus injections to insulin pumps –which provide far greater accuracy as to total daily insulin dose (TDD) - between the 8–12 study visits revealed no statistical differences in IDAA1c between the groups (Supplementary Fig. 8b).

### Effect of GABA alone and GABA/GAD in combination on glucagon

**Fig. 4:**
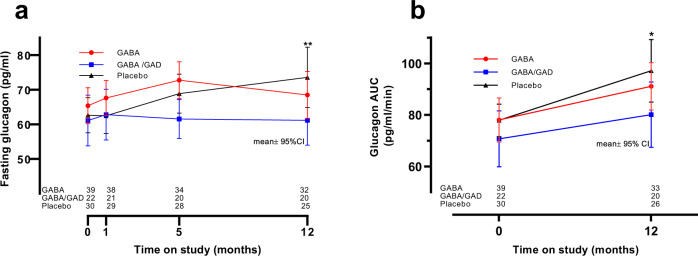
Fasting and AUC glucagon in study groups over time. Fasting glucagon (**a**) was measured in the three study groups (GABA-red, GABA/GAD-blue, and placebo-black) at baseline (Time=0, prior any treatment) and at 1, 5 and 12-months thereafter. AUC glucagon (**b**) was calculated over time in the three study groups. Results are given as mean ±95% CI. Statistical differences were by two-sided analysis of covariance. **a** At 5 month GABA/GAD vrs. GABA, *p* = 0.007 and GABA/GAD vrs. Placebo, *p* = 0.086. At 12- months, GABA/GAD vrs. Placebo,**p = 0.035. Regarding AUC glucagon (**b**), at 12-months GABA/GAD vrs. Placebo, *p = 0.041. Source data are provided as a Source Data file.

As shown in (Fig. 4a), the mean fasting glucagon in the placebo group increased by 16.8% over the course for the study (baseline to 12- months) in contrast to the two study groups wherein this change over time was curtailed: 0.4% in the GABA group and 0% in the GABA/GAD group. At 5 months, the mean fasting glucagon value in the GABA/GAD group was attenuated by 10.7% compared to placebo (*p* = 0.086) and 11.1% compared to the GABA group (*p* = 0.007). By 12-months, the mean fasting glucagon in the GABA/GAD group significantly diminished compared to the placebo patients (*p* = 0.035), but there were no statistical differences relative to the GABA group.

Similar to the fasting glucagon data, the mean area under the curve (AUC) glucagon levels increased from baseline to 12-month in all groups (placebo group (24% increase), GABA group (13.7% increase) and in the GABA/GAD group (13.1%). At 12-months, the AUC glucagon in the GABA/GAD group was significantly reduced compared to placebo (*p* = 0.041) (Fig. 4b).

Based on the association between elevated glucagon and hyperglycemia in T1D^28–32^, we examined the correlation between glucose and glucagon. At first visit (baseline), both fasting glucose (*p* = 0.0017) and AUC glucose (*p* = 0.04) correlated with glucagon (Supplementary Fig. 2a, b**)**. Similar correlations for the 12-month visit were apparent (Supplementary Fig. 2c, d**)**.

### Proinsulin levels and diabetes autoantibody titers

Fasting and 90 min post mixed meal plasma proinsulin and the proinsulin/c-peptide ratio was examined in the three study groups at baseline (before treatment), 5 and 12-months. No differences were detected related to treatment (Supplementary Fig. 3). The time course of diabetes autoantibodies (GAD65, ZnT8, ICA512) is presented in Supplementary Fig. 4a–c. The percent positivity for ZnT8 and ICA512 is presented in Supplementary Fig. 4d. Overall, there were no statistical trends or differences in the diabetes antibodies over time.

### GABA levels

Plasma GABA levels are presented in Supplementary Fig. 5. Participants swallowed their study drug immediately before the mixed meal. There were no differences between the study groups in the 0 min (fasting) GABA levels at either the baseline (initial) or 12-month visit. Not unexpectedly, given the short half-life of GABA, the morning fasting values were statistically the same in all three cohorts. The veracity of the GABA study drug versus placebo is evidenced by the increase in plasma GABA at 60 and 120 min relative to baseline in the GABA and GABA/GAD groups, with no change in the placebo group.

### HLA haplotypes

The primary outcome, fasting and meal-stimulated c-peptide, was re-examined after subdividing each treatment group according to high-risk HLA haplotypes. Results did not show statistically significant differences (Supplementary Table 2).

## Discussion

This prospective, randomized, control trial of GABA and combined GABA/GAD in children with new-onset T1D confirmed the safety and tolerability of oral GABA, but did not attain its primary objective, the preservation of β-cell function (Fig. 2). However, a secondary outcome revealed a significant decrease in fasting, as well as nutrient-stimulated, glucagon secretion following 12-months of oral GABA/GAD treatment (Fig. 4). This observation corroborates favorably with animal/cell studies in which GABA (or GABA/GAD) has a paracrine inhibition on α-cells.

GABA, secreted from β-cells, reportedly has both an autocrine effect on insulin secretion as well as a paracrine inhibition of α-cell glucagon production. Whereas a distinct GABA autocrine role remains unsettled, the physiologically-relevant, paracrine inhibition of glucagon secretion or diminution of α-cell mass has been repeatedly documented in isolated cells or islets, perfused or biopsied pancreata, or in vivo animal studies. Upon binding to its cognate chloride channel, GABA begets α-cell membrane hyperpolarization, thereby hampering voltage-dependent calcium channels, which curtails glucagon output. For example, in streptozotocin (STZ)-induced diabetic mice, 12 days of daily intraperitoneal GABA (10 mg/kg) quenched the robust 7- fold increase in α-cell mass, which occurred in controls. And, relevant to β-cells, GABA augmented the proliferation of α-cells expressing GLP–1. The latter, in turn, could plausibly enhance β-cell function and growth^33^.

In another Type 1 diabetes model (multiple low dose STZ, MDSD), GABA, when added to the drinking water (6 mg/ml), reduced both serum glucagon and α-cell mass^34^. A similar tandem α and β cellular GABA effect was also found in MDSD mice treated with 20 µmol/kg intraperitoneal GABA prior to diabetes induction, and in a series of mice previously rendered diabetic with severe hyperglycemia^19^. Islet studies unfailingly corroborate the inhibitory action of GABA on glucagon secretion. In rat islets, GABA was noted to dampen glucose–stimulated glucagon secretion^35^ and, in normal mice islets or perfused pancreas, an inhibition of glucagon secretion was observed^36^. Finally, when a non-curative mass of normal human islets was transplanted into diabetic mice (NOD – seid- ϒ), after 5 weeks of drinking water with GABA added (6 mg/ml), serum glucagon was reduced roughly 80%^37^.

Antagonism of the glucagon receptor, or by genetic knockout, especially in the face of insulin deficiency, promotes normoglycemia. Take, for example, the following observations: (i.) Even without supplemental insulin, by blocking the glucagon receptor in diabetic obese mice, hyperglycemia was normalized^38^, (ii.) In the high-fat type 2 diabetic mouse, knockout of the glucagon receptor aborted obesity, hyperinsulinemia and abnormal lipogenesis and, notably, prevented hyperglycemia^39^, (iii.) In glucagon receptor null mice, following massive streptozotocin β−cell destruction, and despite marked hyperglucagonemia (14-fold increase over wild-type), normal blood glucose prevailed^40^, (iv.) Glucagon receptor antibody alone, i.e., no insulin therapy, can normalize hyperglycemia of type 1 diabetic NOD mice^41^, and finally, (v.) In humans with T1D, a single subcutaneous dose of a glucagon receptor antibody resulted within days in a 14% reduction in insulin dose and improved glycemic control as assessed by continuous glucose monitoring^31^. Most recently, a monoclonal glucagon receptor antagonist (Ab-4) corrected both glycemia and provoked restoration of β−cells in type 1 diabetic rodents (NOD and PANIC-ATTAC mouse models) as well as in mouse-implanted human islet xenografts^42^. Indeed, in the NOD mouse, the Ab-4 antibody increased insulin islet area approximately 900% versus control.

In concordance with previous reports, we found a progressive increase in serum glucagon over the first year following T1D diagnosis (Fig. 4), a phenomena which can persist for 3–5 years^43–47^. Glucagon may worsen glycemic control^28,29^ by peripheral effects on hepatic, adipose, and neural metabolism. Even in non-diabetic adults, fasting glucagon correlates inversely with longitudinal β−cell function- inferring that α-cell dysfunction is an incipient stage in disturbed glucose metabolism^48^. Although suppression in serum glucagon by GABA/GAD was found in our study, the percent lowering may not be sufficient to impact glycemic control (Fig. 3), namely, the insulin-adjusted A1c (IDAA1c) in this group. Of interest, using the reference standard for IDAA1c, a meal stimulated c-peptide >300 pM, there was a trend suggesting improvement in GABA/GAD group at 5-months (Supplementary Fig 8a). As evidenced in Supplementary Fig. 2, serum glucagon correlates positively with serum glucose, which infers a role of glucagon in glucose homeostasis. In our placebo cohort, the AUC glucagon at one year was 24% elevated versus baseline. This compares favorably to the postprandial increases of 37 and 51% previously reported in children with T1D (references^45^ and^43^, respectively).

The slight increase in IDAA1c in the GABA group (Fig. 3b) warrants discussion. This calculated metric of glycemic control^27^ is the least objective index insofar as it incorporates TDD, which in our study depended on participant paper records and recall. Furthermore, TDD is influenced by exercise, carbohydrate load, intercurrent illness and other factors. As aforementioned, using a meal-stimulated c-peptide >300 pmol/l^27^, there was no difference in glycemic control between the three groups (Supplementary Fig. 8a). Likewise those patients who transitioned to insulin pumps, which provide a more precise digital assessment of TDD, showed no differences in IDAA1c (Supplementary Fig. 8b).Finally, fasting and AUC glucose were not different amongst the groups at 12 months (Supplementary Fig. 9).

This study has many strengths. Foremost, as an adjunct agent, it is the first GABA study conducted in newly diagnosed humans. Further, by studying an exclusively pediatric population, we were able to enroll very young patients with T1D who typically have a more rapid decimation of β cells than adolescents^49–53^. Forty percent of our study participants were <10 years. Considering the array of confounding factors in β-cell loss, age of onset is the major determinant in the temporal decline in serum c-peptide. Most other potential therapeutics are first investigated in the adult population, making it impossible to reliably exclude patients with latent autoimmune diabetes of adulthood (LADA)^54^. Thirdly, our study was able to enroll all children within the first 5 weeks after diagnosis, allowing exposure of the pancreatic islets to the intervention before near-total autoimmune β-cell eradication.

Our study established that oral GABA is tolerable. The basis for this “low-dose” designation merits consideration. The daily dose of GABA used in animal studies, mostly mice, are sweeping, ranging 0.25 mg to 1500 mg/kg. Under FDA constraints, our dose of 1 gram/M^2^ (about 35 mg/kg) was far below nearly all in vivo studies in which salutary outcomes were reported (Supplementary Fig. 6).

It is speculative as to the mechanism whereby the GABA/GAD tandem attenuated glucagon more than GABA alone. However, the combination GABA/GAD strikingly extended, and in a synergistic manner, the time to develop hyperglycemia in diabetic NOD mice with transplanted β-cells^25^. It is conceivable that GAD-alum may have increased ambient islet cell insulin concentrations - despite no detectible change in the systemic serum levels - thereby reducing adjacent alpha cell glucagon release. To the point, we could have included a GAD-alum group alone, however, we did not because of the previous single and multicenter GAD-alum studies^14,15,55–57^.

Proinsulin and the proinsulin/c-peptide ratio are recognized markers of β-cell stress in T1D, likely related to aberrant proinsulin processing^58,59^. We investigated whether proinsulin or the proinsulin/c-peptide ratio was modified by treatment with GABA, or the combination GABA with GAD, due to their recognized immunosuppressive actions in diabetes^25^ (Supplementary Fig. 3), no statistical differences were identified. Likewise, there was no difference in baseline or subsequent diabetes antibody titers or positivity in the treatment groups (Table 1 and Supplementary Fig. 4) which is not unexpected for a one-year T1D trial^60^.

Considering the role of the DR3-DQ2 haplotypes which confer T1D risk and disease course^61^, we screened our study cohorts accordingly. Based on previous evidence demonstrating HLA haplotype specificity to GAD-alum therapy^16^, we examined whether the presence or absence of HLA DR3-DQ2 altered the primary outcome in the three treatment groups. No differences were detected; however, a larger cohort may be required to detect statistical distinctions (Supplementary Table 2).

The study has limitations, most notably the unpropitious compliance (assessed by pill counts and recall,) as is commonly encountered in the real-clinic setting (Supplementary Fig. 7). Based on in vivo animal trials, the dose of GABA (alone) may have been inadequate, namely, beneath a threshold response (Supplementary Fig. 6). As aforementioned, a further weakness of our study was that the GABA preparation was relatively short acting and taken only twice daily (Supplementary Fig. 5). Alternatively, long-acting preparations of GABA and/or currently available GABA-ergic drugs that have longer half-lives of action offer promise. And, based on affirmative β-cell studies in human islets, co-treatment of GABA with an allosteric positive modulator (Ly49) of its cognate receptor is an ingenious notion^62^.

To sum, in this prospective, randomized controlled trial of twice-daily GABA, or co-treatment with GABA/GAD, in humans with T1D, we demonstrate a significant decrease in fasting and AUC glucagon in the GABA/GAD group, with a non-significant reduction in the GABA group at 12-months. There were no statistically significant changes in the primary outcome, namely, fasting and meal-stimulated c-peptide between the cohorts. Notwithstanding the necessarily low GABA dose for this trial in TID children, in combination with the compliance challenge, the reduction in serum glucagon augurs well for further studies to conceivably preserve β-cell function or mass. Indeed, in the sole study using co-therapy with GABA/GAD, β-cell preservation was dependent on the dose^25^. Lastly, bearing in mind that GABA/GAD attenuated glucagon production, this could in turn expand β-cell mass and/or improve glucose homeostasis. Case in point, in diabetic mice, blocking glucagon action begets a nearly 8-fold increase in insulin-positive islet cell mass and mediates β-cell regeneration^42,63^. Insofar as GABA tempers immune inflammation at higher doses in rodents, and our study was constrained to relatively low-dose GABA dosing in this pediatric trial in T1D, it is plausible that increased GABA doses, or long-acting preparations, could offer sufficiently prolonged, above-threshold GABA concentrations to preserve islet cells, particularly during stage 1 diabetes.

## Methods

The detailed rationale and methods for this study have been described elsewhere with minor modification^26^. A succinct summary follows:

### Study design and treatment

This is a prospective, one-year randomized, double blind, placebo controlled trial to evaluate the safety and efficacy of GABA alone and combination GABA/GAD-alum® in children with newly diagnosed T1D (https://clinicaltrials.gov/ct2/show/NCT02002130). Patients were randomized into one of three study arms (Fig. 1). The original clinicaltrial.gov posting (2013) predates the final protocol submission (2015). We had a protracted period (2 years) prior to study launch in order to obtain FDA approval to administer GABA in children (first human trial). The formal study protocol approvals and funding were in place by 2015 and the first patient enrolled 3/2/2015. Suboptimally, we noticed the documentation discordance from 2013 and updated the clinicaltrial.gov outcomes in July 2019 to align with the 2015 study protocol.

### Participants and eligibility criteria

Participants were screened at the time of diagnosis with T1D, as defined by ADA criteria. All patients were enrolled from the clinics and in-patient wards at Children’s of Alabama (CoA), a tertiary care university-associated referral center. The majority of patients were residents of the state of Alabama. There were 11 out of state participants (AZ, GA, MS, MO, NC, ND, TX, VA). The first participant was enrolled 3/2/2015 and the last study visit was 6/24/2019. Inclusion criteria: children 4–18 years of age, positivity for autoantibody GAD65, and enrollment within 5 weeks of diagnosis. If the participant was female and not abstinent, two forms of contraception were required. Exclusion criteria: pregnancy, systemic or inhaled steroid use, neurologic/seizure disorders, adjunct oral therapies that might affect glucose or GABA metabolism^26^. Six randomized patients were excluded from analysis because all c-peptide values, fasting and MMTT stimulated, were <0.6 ng/ml at the initial baseline study visit^64,65^.  Participants received a $60 gift card as compensation for every blood draw.

### Randomization

Patients were randomized into one of three regimens (GABA, GABA/GAD-alum, or placebo) stratified by age and in balanced blocks of three (1:1:1) for the first 75 patients using a pre-set randomization list (generated by using a computerized procedure) known only to the un-blinded pharmacist. This second protocol was a consequence of unanticipated additional funding that afforded trial extension for the GABA versus placebo groups only.

### Study drugs

#### Oral gamma-aminobutyric acid (GABA)

GABA and placebo capsules were prepared commercially (NOW Foods, Bloomingdale, IL). GABA or placebo was administered using premeasured capsules (1 gram/M2/day up to maximum of 1.5 gram/day) divided into two daily doses (morning and evening). The purity of both the GABA and placebo products was verified by LC/MS/MS prior to study enrollment. The control GABA that was used for mass spectroscopy analysis was obtained from Sigma-Aldrich Chemical Company (St. Louis, MO). Placebo and GABA capsules were, taste-wise and visually, indistinguishable.

#### Glutamic acid decarboxylase (GAD-alum)

GAD-alum and placebo were prepared as a suspension with recombinant GAD enzyme and the vaccine adjuvant Alhydrogel ® (alum) by Diamyd Medical (Stockholm, Sweden). The subcutaneous GAD-alum injections (20 μg/dose), or placebo, were given in clinic by the research nurse.

### Mixed meal tolerance testing (MMTT)

MMTT occurred according to the visit schedule outlined in Table 2 and as described previously^26^.

**Table 2:** Study treatment visit schedule

			Study visit				
Study group	Treatments		Baseline Visit #1	Month 1 Visit #2	Month 5 Visit #3	Month 8 Visit #4	Month 12 Visit #5
GABA	GABA oral twice daily	Placebo-GAD one injection: visits #1 and #2	MMTT^a^	MMTT	MMTT	HbA1c Insulin- dose	MMTT
GABA/GAD^b^	GABA oral twice daily	GAD one injection: visits #1 and #2	MMTT	MMTT	MMTT	HbA1c Insulin- dose	MMTT
Placebo	Placebo-GABA oral twice daily	Placebo-GAD one injection: visits #1 and #2	MMTT	MMTT	MMTT	HbA1c Insulin- dose	MMTT

^a^MMTT- Mixed meal tolerance test. ^b^GAD = GAD-alum (Diamyd, Stockholm, Sweden) was administered on visit #1 and the second dose was administered on visit #2.GABA (gamma aminobutyric acid), insulin-dose (total daily insulin dose).

### Safety monitoring

Safety assessments included observations of reactions at the injection site, occurrence of all adverse events (AEs)/serious adverse events (SAEs), laboratory measurements (chemistry panel, complete blood counts with differential, and urinalysis), neurological assessments, and physical examination.

### Adherence and retention measures

Treatment adherence of the oral capsules was assessed subjectively by patient recall, and objectively by calculating the unused capsule count at each visit. Study participants were asked to return any unused study drug for safe disposal and queried whether any capsules were destroyed or lost.

### Investigative endpoints

The primary outcome measure was the effect of GABA or GABA/GAD on fasting and meal-stimulated serum c-peptide compared to placebo at baseline,1-month, 5-months and 12-months. Secondary endpoints included (1) fasting and meal-stimulated glucagon and proinsulin (2) glycemic control (HbA_1c_, IDAA1c^27^), (3) diabetes autoantibodies, and (4) immune studies in peripheral blood mononuclear cells (to be presented in a separate manuscript). Exploratory endpoints included plasma GABA levels and the proinsulin/c-peptide ratio before and after meal-stimulation. Also, we examined the effect of diabetes-related HLA risk haplotype on the primary outcome.

### Endocrine assays

C-peptide, glucose and glucagon were measured in the University of Alabama Core Metabolic Laboratory and as previously noted^26^. C-peptide was measured by a two-site immunoenzymometric analyzer (900 AIA-Pack, TOSOH, San Francisco, CA) and glucagon by radioimmunassay (Millipore Sigma, Burlington, MA). Antibodies to GAD65, IA512, and Zinc 8 Transporter were assayed commercially by Labcorp (Burlington, NC) as standard of care.

### Plasma GABA

Plasma GABA levels were obtained during mixed meal tolerance test (MMTT) at both the baseline (initial study visit) and 12 month visits. Patients swallowed oral study drug dose at 0 min, immediately prior to ingesting mixed meal drink. GABA levels were determined at 0, 60 and 120 min.

### GABA analysis

#### Materials and sample preparation

Solid stocks of GABA and GABA-d6 were purchased from Sigma & CDN Isotopes respectively. Standards were reconstituted in methanol. The analytical range was 1–5000 ng/ml over 8 calibrators. Plasma samples were thawed on ice and spiked with 10 µl of 500 ng/ml GABA-d6. They were transferred quantitively to 1 cc Phree SPE cartridges (Phenomenex, Torrance, CA) containing 600 µl of 1% formic acid acetonitrile, incubated at room temp for 5 min and centrifuged at 1000 *g* for 5 min. The flow-through was retained and transferred to a Biotage N2 evaporator to dry. Samples and standards were reconstituted in 100 µl of 1.0% formic acid before analysis. *LC-MS Conditions*. Separation and detection were carried out by Shimadzu Prominence 20 series HPLC in tandem with a Sciex API 4000 triple quadrupole mass spectrometer (MS) utilizing a modified method from Imtakt^66^. Chromatographic separation occurred with a Intrada Amino Acid column 3 µM 50 ×3 mm at 60 degrees C. Mobile phases were A) 0.3% formic acid in MeCN and B) 100 mM ammonium formate. Gradient schedule was as follows: 0 min 30% B, 4 min 35% B, 5 min 100% B, 5.1 min 30% B, and 5.5 min stop. The flowrate of 0.6 ml/min. Injection volume was 5 µl. Analyst v1.7.2 was used for instrument control & data acquisition. The MS was operated in positive polarity electrospray ionization. MS source parameters were as follows: collision gas 5, curtain gas 25, GS1 40, GS2 45, IS 2000, and temperature 600. Compound mass transitions were 104.1 m/z à 87 m/z & 110 m/z à 93 m/z for GABA and GABA-d6 respectively. Compound parameters were as follows: collision energy 15, cell exit potential 6, and declustering potential 60. Data processing occurred in MultiQuant v3.0.3. The standard curve was regressed linear with 1/x2 weighting

### HLA genotyping in study participants

The Histocompatibility and Immunogenetics Laboratory at the University of Alabama at Birmingham performed HLA typing on genomic DNA that was isolated from frozen peripheral blood mononuclear cells (PBMC).

### Statistical analysis

Baseline demographic and other clinical characteristics were compared between the treatment groups using *t*- and chi-square tests (or their non-parametric equivalents) for continuous and categorical variables, respectively. Analysis of covariance was used to compare changes in C-peptide levels between the treatment groups. For these analyses, the 12-month measurement served as the dependent variable with two independent variables: (1) a categorical variable for treatment group and (2) the baseline C-peptide measurement. A similar analytical approach was used for the other study outcomes of interest including glucagon, hemoglobin A1C, IDAA1C, and total daily insulin dose. Mixed statistical models were used to conduct longitudinal analyses of C-peptide and hemoglobin A1C measurements, and daily insulin requirements, incorporating all three measurements. This study utilized REDCap (Research Electronic Data Capture, version 12.3.3 https://www.project-redcap.org), a software toolset and workflow methodology for electronic collection and management of clinical and research data. Data analysis of for primary, secondary and exploratory outcomes used SAS/STAT software, version 9.4 of the SAS System. Copyright, SAS Institute Inc. Cary, NC, USA. Graphs were prepared with GraphPad Prism 9.0 for Windows, GraphPad Software, San Diego, CA, USA, www.graphpad.com. Correlations and Fisher’s exact analyses were by GraphPad.

### Reporting summary

Further information on research design is available in the Nature Portfolio Reporting Summary linked to this article.

## Supplementary information

Supplementary Information

Reporting Summary

## Data Availability

Following de-identification, all of the individual participant data collected during this trial, as well as data dictionaries, will be available to any researcher who provides a methodologically-sound proposal for academic purposes. Requests should be directed to the corresponding author and is subject to a material transfer agreement. Proposals may be submitted up to 36-months following publication. Source data are provided with this paper. The study protocol is available online (https://clinicaltrials.gov/ct2/show/NCT02002130). Source data are provided with this paper.

## Source data

Source Data
