# Supplementary material for: A randomized trial of oral gamma aminobutyric acid (GABA) or the combination of GABA with glutamic acid decarboxylase (GAD) on pancreatic islet endocrine function in children with newly diagnosed type 1 diabetes
Source: Nat Commun. 2022 Dec 24;13:7928. doi: 10.1038/s41467-022-35544-3 (PMC9790014; doi:10.1038/s41467-022-35544-3)
Supplement: Supplementary file 1 — Supplementary Information [file 41467_2022_35544_MOESM1_ESM.pdf]

Supplementary Information

Supplementary Figures 1-9 and Tables 1-2

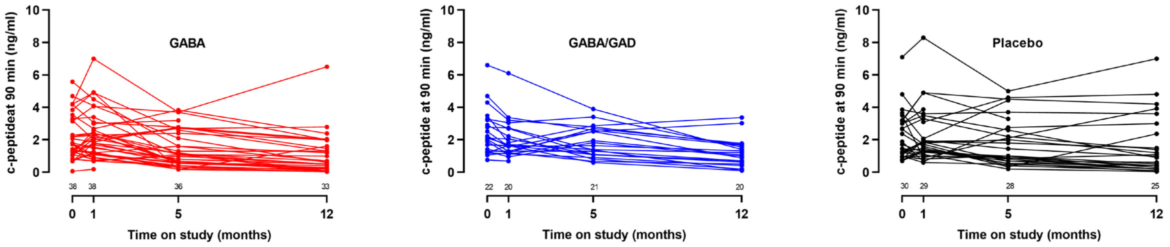

Supplementary Figure 1. Effect of GABA alone and combination GABA/GAD on c-peptide over time. Serum c-peptide at 90 minutes post mixed meal in children with new-onset T1D treated for one year with GABA, GABA/GAD or placebo. The number (n) of study participants at each time point is noted above the x-axis. Source data are provided as a Source Data file.

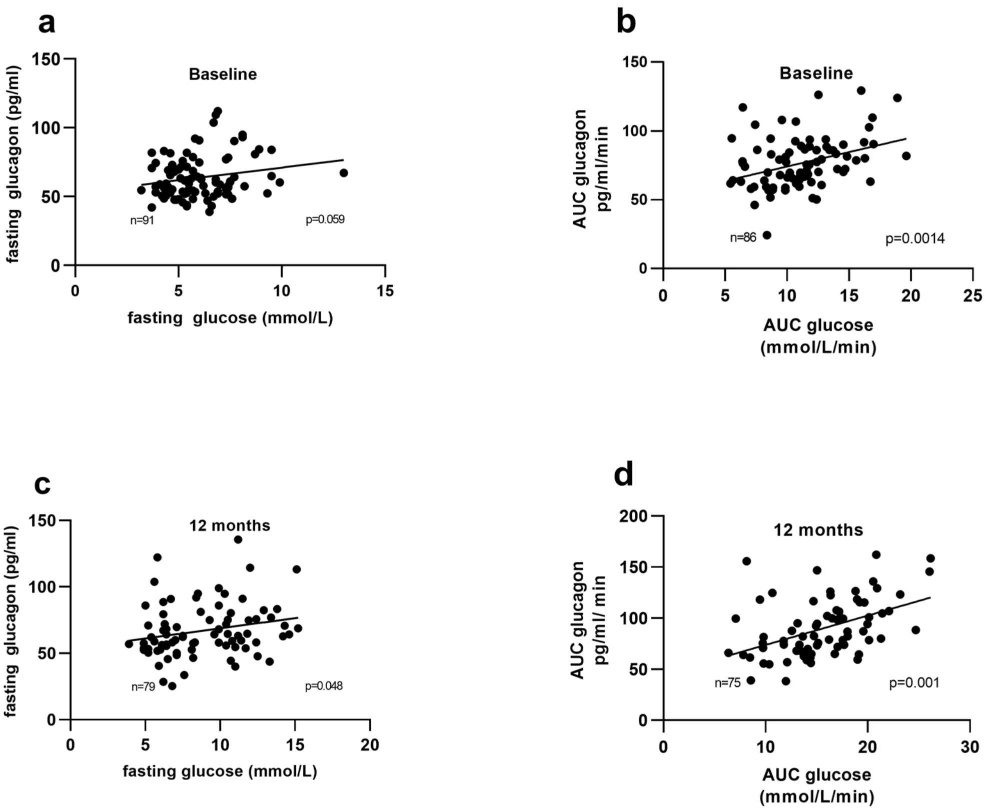

Supplementary Figure 2. Correlation between glucagon and glucose at baseline and 12 months. (**a**) Baseline fasting glucagon versus fasting glucose, (**b**) 12 month fasting glucagon versus fasting glucose, (**c**) Baseline AUC glucagon versus AUC glucose, (**d**) 12 month AUC glucagon versus AUC glucose. All study participants were combined for each figure. Statistical p values were derived by linear regression. Analysis was by GraphPad Prism 9.0 (GraphPad Software, San Diego, CA, USA, www.graphpad.com. Source data are provided as a Source Data file.

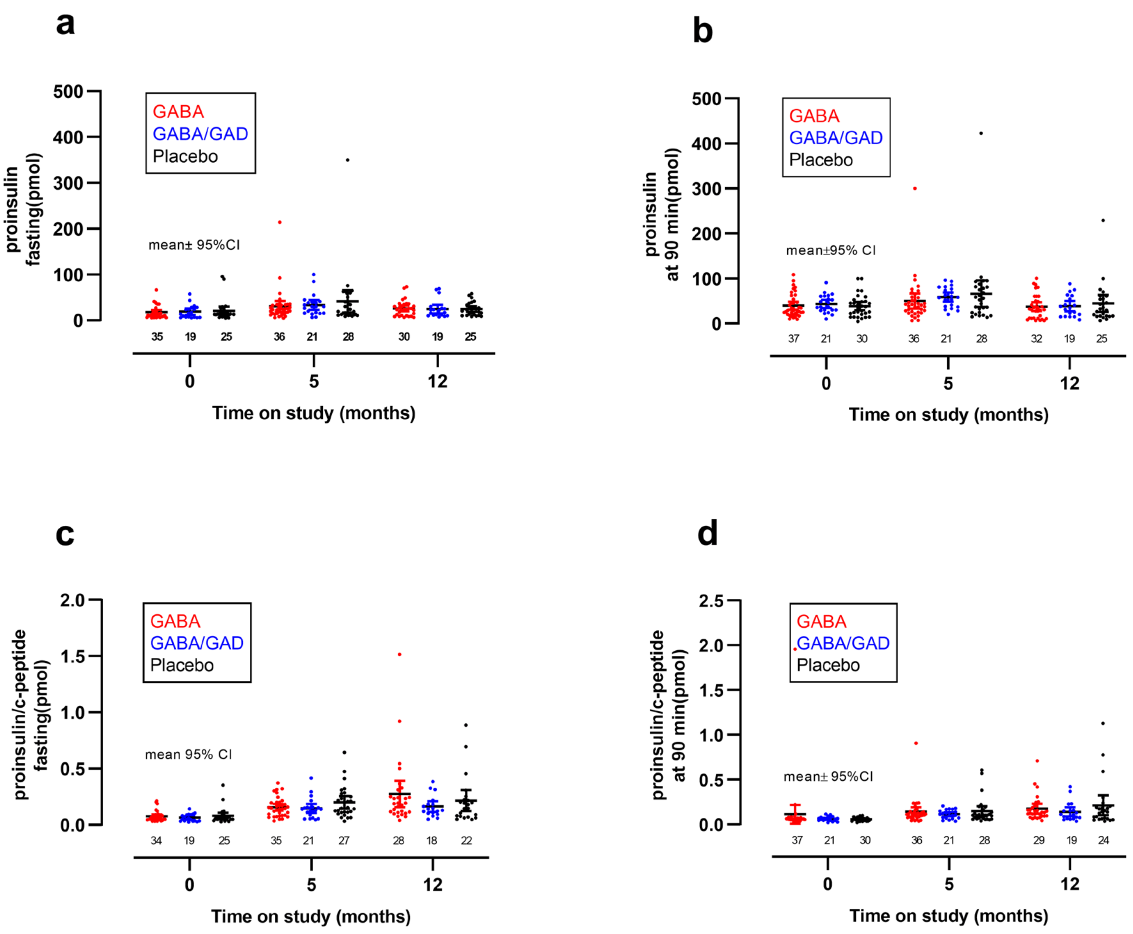

Supplementary Figure 3. Proinsulin and proinsulin/c-peptide ratio in study groups. (**a**) Fasting proinsulin, (**b**) 90min post mixed meal tolerance test (MMTT) proinsulin, (**c**) Fasting proinsulin/c-peptide and (**d**) 90 minutes post MMTT proinsulin/c-peptide. No statistical differences were identified between the groups. Analysis was by two-way analysis of variance. The number (n) of study participants at each time point is noted above the x-axis. Results are presented as mean 95%CI. Source data are provided as a Source Data file.

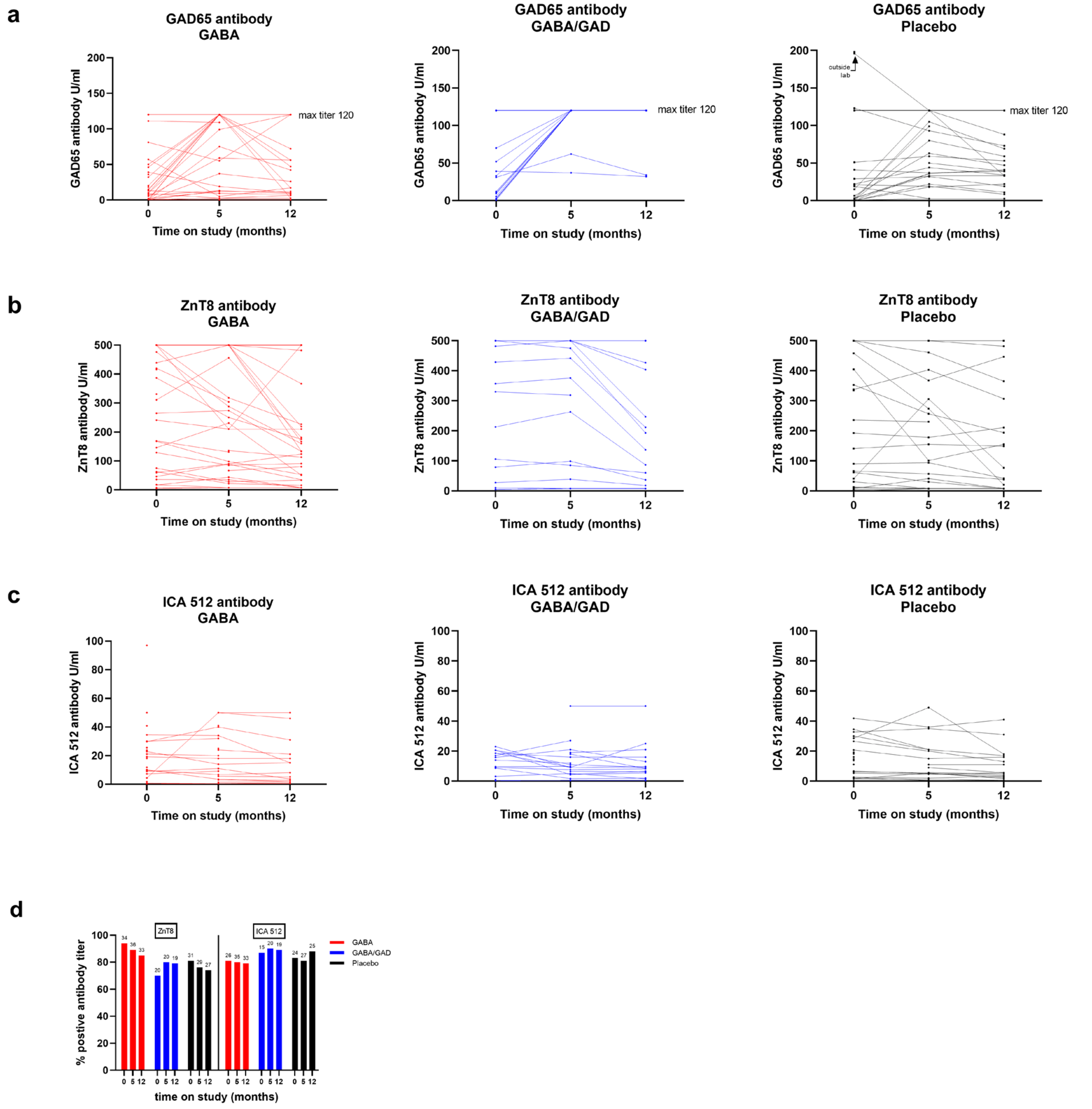

Supplementary Figure 4. Diabetes antibody titers in study patients at baseline, 5 months and 12 months. Three antibodies were measured (**a-c**) with positive titer cut-offs as follows: (**a**) GAD65 (>0.5 U/ml), **(b)** ZincT8 (>15 U/ml) and **(c)** ICA 512 (>1 U/ml). The assay normal range for ICA512 (**c**) changed mid-study to a positive titer being >15 U/ml. This affected 35 samples (20 GABA, 15 GABA/GAD and 12 Placebo at the 5 and 12 months visits) hence these values are not included in supplementary Fig 4**c**. Overall antibody titer positivity is presented in Fig 4**d** that includes all patient data regardless of normal range. The number atop each bar (**d**) indicates the number of patient samples included. There were no statistical differences in antibody positivity over time. Statistical comparisons were by two-way analysis of variance. Source data are provided as a Source Data file.

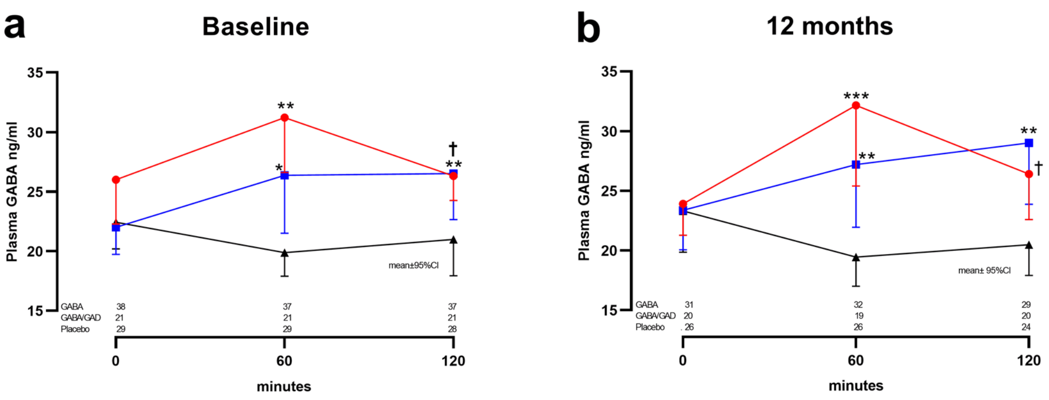

Supplementary Figure 5. Plasma GABA levels during mixed meal tolerance test (MMTT) at baseline (initial study visit) and 12 month visits. Patients swallowed oral study drug dose at 0-minutes, immediately prior to ingesting the mixed meal drink. GABA levels were determined at 0, 60 and 120 minutes as per Methods. Red line = GABA, blue line = GABA/GAD and black line = placebo. Results are expressed as mean ± 95%CI. Statistical differences were assessed by two-tailed Mann Whitney test, using GraphPad Prism 9.0 (GraphPad Software, San Diego, CA, USA, www.graphpad.com. (**a**) Baseline statistical differences at 60 minutes:** p =.005 GABA vrs. Placebo and*p =.030 GABA/GAD vrs. Placebo. At 120 minutes: **p =.005 GABA vrs. Placebo and †p = .010 GABA/GAD vrs. Placebo. (**b**) 12 month statistical differences at 60 min: *** p =.001 GABA vrs. Placebo and **p =.005 GABA/GAD vrs. Placebo. At 120 minutes: **p =.005 GABA vrs. Placebo and †p = .01 GABA/GAD vrs. Placebo. The number (n) of study participants at each time point is noted above the x-axis. Source data are provided as a Source Data file.

.

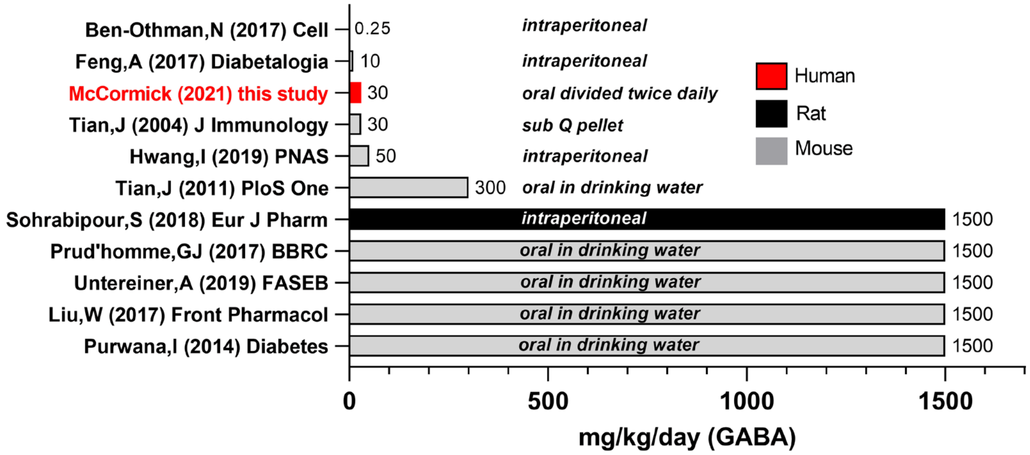

Supplementary Figure 6. Summary of Experimental GABA doses. When GABA was added to drinking water, as was done in many rodent studies, the daily intake approximated 1500 mg/kg based on estimated daily water consumption[1]. This calculation does not take into account that diabetic animals have polydipsia, so the actual GABA dose likely exceeds 1500 mg/kg per day. Allometric scaling of the GABA dose to body surface area closed the gap between our dose in children versus other vertebrates[2]. Using the "Km factors" of 3 and 25 for the mouse and children, respectively, the daily dose in our study of 1 gram/M^2 is equivalent to 250 mg/kg in mouse experiments[3,4]. Ideally, given the half-life and time to peak found in our pharmacokinetic study in healthy adults (not published) and in one published study[5], oral GABA should preferentially be taken more than twice daily. For this first human trial of oral GABA in children, we reasoned that twice daily dosing would make optimal compliance more attainable. Figure references: Ben-Otherman, N (2017) Cell[6]; Feng, A. (2017) Diabetalogia[7]; Tian, J (2004) J Immunology[8]; Hwang, I (2019) PNAS[9]; Tian, J (2011) Plos One[10]; Sohrabipour, S (2018) Eur J Pharm[11]; Prud'homme, GJ (2017) BBRC[12]; Untereiner, A (2019) FASEB[13]; Liu, W (2017) Front Pharmacol[14]; Purwana, I (2014) Diabetes[15].

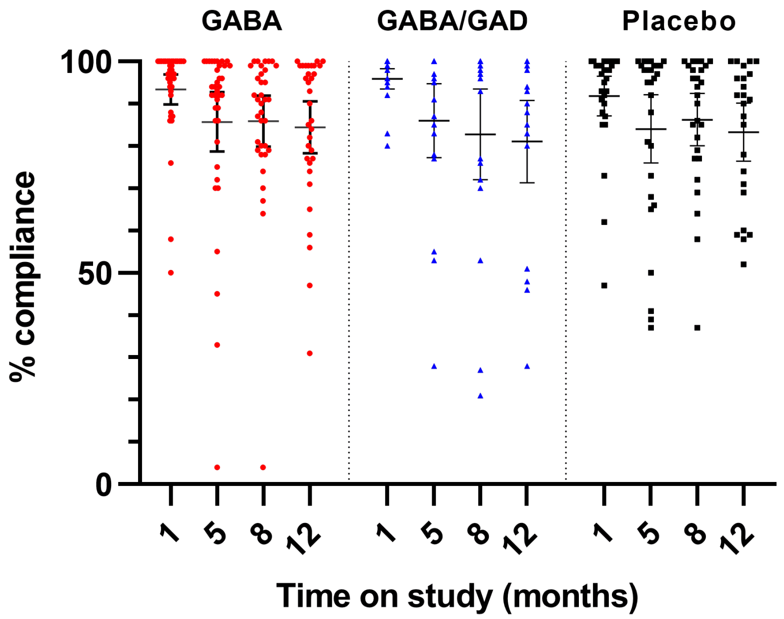

Supplementary Figure 7. Patient compliance with oral GABA treatment. Treatment adherence of the oral capsules was assessed subjectively by patient recall, and objectively by calculating the unused capsule count at each visit. The percent compliance for a particular interval was the number of capsules returned/number of capsules dispensed times 100. The mean (95%CI) is shown as bar in each scatter dot plot using GraphPad Prism 9.0 (GraphPad Software, San Diego, CA, USA, www.graphpad.com At 12 months, these values were: GABA 84.4 (78.3,90.6) n=33, GABA/GAD 81.1 (71.3,90.8) n=20, Placebo 83.3 (76.4,90.2) n=24. There were no statistical differences in compliance by two-way analysis of variance. Source data are provided as a Source Data file.

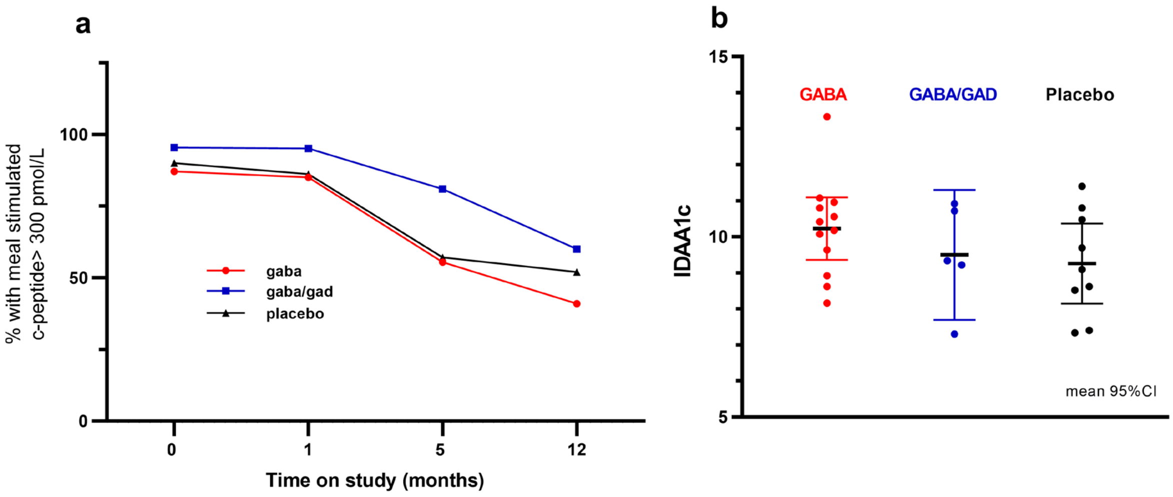

Time on study	GABA vrs Placebo	GABA/GAD vrs Placebo	GABA vrs GABA/GAD
0 month (baseline)	>0.999	>0.999	0.629
1 month	>0.999	0.383	0.404
5 months	0.999	0.124	0.084
12 months	0.428	0.676	0.255
statistical comparisons by Fisher's exact test.			

Supplementary Fig. 8. Alternative measures of glycemia in study groups.
Two metrics of glycemic control were analyzed that do not depend on written home records to determine total daily insulin dose (TDD). (**a)** Reference standard index for IDAA1c. Using the gold standard reference for IDAA1c, namely, a meal stimulated c-peptide >300 pM[16], the percent of study participants in each group with a c-peptide >300 pM is plotted at baseline, 1 month, 5 months and 12 months. Statistical differences by the Fisher's exact test are presented in the table below Fig 3a. (Analysis was by GraphPad Prism 9.0 (GraphPad Software, San Diego, CA, USA, www.graphpad.com). (**b)** IDAA1c in participants on insulin pumps at 12 months**.** About 30% of study participants (36% GABA (n=12), 24% GABA/GAD (n=5) and 36% Placebo (n=9)) transitioned from basal/bolus regimens to insulin pumps between 8 and 12 months. Data extracted from insulin pumps is more accurate than injected basal/bolus paper records or recall concerning the TDD calculation. Analysis of IDAA1c in this subgroup of participants using pump data at 12 months (mean ± 95%CI) revealed no statistical differences by two-way analysis of variance ($p=0.174$). Source data are provided as a Source Data file.

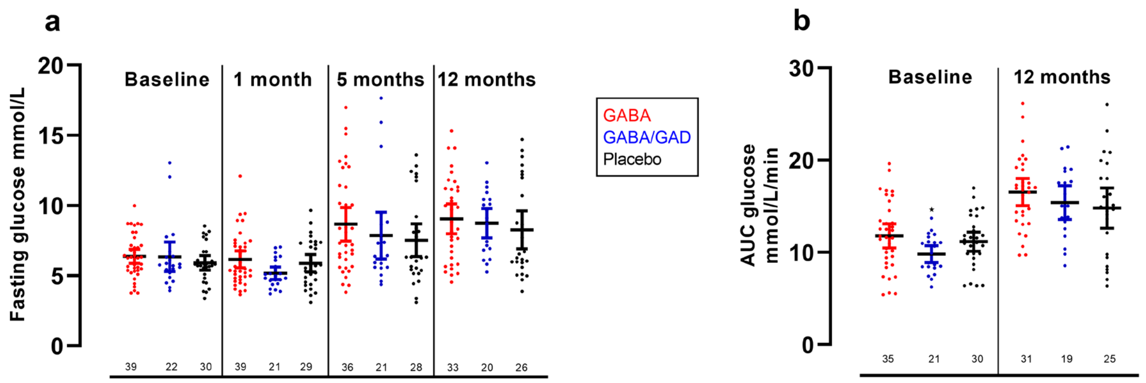

Supplementary Fig. 9 Fasting and AUC glucose in the study groups. Fasting glucose (**a)** was measured in the three study groups (GABA-red, GABA/GAD-blue, and placebo-black) at baseline (Time = 0, prior any treatment) and at 1, 5 and 12 months thereafter. Area under the curve (AUC) glucose (**b**) was measured at baseline and 12 months in the three study groups. There were no statistical differences in fasting glucose (9a) between the groups by two-way analysis of covariance. Regarding AUC glucose (9b), at baseline, the GABA/GAD group was 17% reduced compared to the GABA group (*p=0.0172) but was not different from placebo (p=0.351). At 12 months, there were no statistical differences in AUC glucose between the groups. Results are presented as mean ± 95% CI. The number (n) of study participants at each time point is noted above the x-axis. Source data are provided as a Source Data file.

Supplementary Table 1: Statistical differences for primary and secondary outcomes

Parameter/visit	mean [95% CI LL-UL]			p values			Difference between means [95%CI LL-UL]		
**Fasting c-peptide**	**GABA**	**GABA/GAD**	**Placebo**	**GABA vrs Placebo**	**GABA vrs GABA/GAD**	**GABA/GAD vrs placebo**	**GABA vrs Placebo**	**GABA vrs GABA/GAD**	**GABA/GAD vrs placebo**
Baseline	0.74 [95% CI 0.57- 0.92]	0.78 [95%CI 0.54 - 1.01]	0.72 [95%CI 0.52 - 0.93]	0.886	0.810	0.724	0.02 [95%CI -0.25- 0.29]	*-0.03 [95%CI -0.33 - 0.26]	0.06 [95%CI -0.25 - 0.36]
1 month	0.80 [95% CI 0.62 - 0.97]	0.81 [95% CI 0.57 - 1.05]	0.82 [95%CI 0.62 -1.02]	0.872	0.930	0.956	*-0.02 [95%CI -0.29 - 0.25]	*-0.01 [95%CI -0.31 - 0.29]	*-0.01 [95%CI -0.32 - 0.31]
5 months	0.68 [95% CI 0.51 - 0.85]	0.76 [95% CI 0.53 - 0.98]	0.66 [95% CI 0.47 - 0.86]	0.917	0.577	0.534	0.01 [95%CI - 0.24- 0.27]	*-0.08 [95%CI - 0.36 - 0.20]	0.09 [95%CI -0.20 - 0.4]
12 months	0.45 [95%CI 0.29 - 0.62]	0.50 [95%CI 0.28 - 0.71]	0.57 [95%CI 0.38 - 0.76]	0.373	0.745	0.633	*-0.11 [95%CI -0.36 - 0.14]	*-0.04 [95%CI -0.32 - 0.23]	*-0.07 [95%CI -0.35 - 0.22]
**AUC c-peptide**	**GABA**	**GABA/GAD**	**Placebo**	**GABA vrs Placebo**	**GABA vrs GABA/GAD**	**GABA/GAD vrs placebo**	**GABA vrs Placebo**	**GABA vrs GABA/GAD**	**GABA/GAD vrs placebo**
Baseline	1.85 [95% CI 1.46 - 2.24]	2.13 [95%CI 1.61 - 2.65]	1.87 [95%CI 1.42 - 2.31]	0.954	0.633	0.448	*-0.02 [95%CI -0.61- 0.58]	*-0.28 [95%CI -0.93 - 0.37]	0.26 [95%CI -0.42 - 0.95]
12 months	0.94 [95%CI 0.52 - 1.37]	1.05 [95%CI 0.50 - 1.60]	1.41 [95%CI 0.93 - 1.89]	0.155	0.752	0.338	*-0.47 [95%CI -1.11 - 0.18]	*-0.11 [95% -0.81 - 0.59]	*-0.35 [95%CI -1.09 - 0.38]
**Fasting glucagon**	**GABA**	**GABA/GAD**	**Placebo**	**GABA vrs Placebo**	**GABA vrs GABA/GAD**	**GABA/GAD vrs placebo**	**GABA vrs Placebo**	**GABA vrs GABA/GAD**	**GABA/GAD vrs placebo**
Baseline	65.35 [95% 60.45 - 70.26]	61.11 [95%CI 54.58 - 67.63]	62.62 [95%CI 57.03 - 68.21]	0.467	0.304	0.727	2.73 [95%CI -4.70 - 10.17]	4.25 [95%CI -3.91 - 12.41]	*-1.51 [95%CI -10.11 - 7.08]
1 month	67.61 [95%CI 62.84 - 72.37]	62.78 [95%CI 56.28 - 69.28]	62.50 [95% 56.97 - 68.03]	0.168	0.727	0.947	5.11 [95%CI -2.19 - 12.41]	4.82 [95%CI -3.24 - 12.89]	0.28 [95%CI -8.25 - 8.82]
5 months	72.71 [95%CI 67.85 - 77.58]	61.53 [95%CI 55.09 - 67.96]	68.88 [95%CI 63.45 - 74.32]	0.299	0.007	0.086	3.83 [95%CI -3.46 - 11.12]	11.19 [95%CI 3.12 - 19.25]	*-7.36 [95%CI -15.78 1.07]
12 months	68.50 [95%CI 61.00 - 74.00]	61.15 [95%CI 52.80 - 69.50]	73.15 [95%CI 65.83 - 80.48]	0.346	0.171	0.035	*-4.66 [95%CI -14.45 - 5.13]	7.35 [95%CI -3.23 - 17.93]	*-12.01 [95%CI -23.11 - 0.90]
**AUC glucagon**	**GABA**	**GABA/GAD**	**Placebo**	**GABA vrs Placebo**	**GABA vrs GABA/GAD**	**GABA/GAD vrs placebo**	**GABA vrs Placebo**	**GABA vrs GABA/GAD**	**GABA/GAD vrs placebo**
Baseline	78.05 [95%CI 70.68 - 85.43]	70.73 [95%CI 60.91 - 80.55]	77.90 [95%CI 69.50 - 86.31]	0.979	0.239	0.273	0.15 [95%CI -11.04 - 11.33]	7.32 [95%CI -4.96 - 19.60]	*-7.18 [-20.10 - 5.75]
12 months	91.13 [95%CI 81.57 - 100.70]	80.11 [95%CI 67.82 - 92.39]	97.13 [95%CI 86.36 - 107.90]	0.410	0.162	0.041	*-5.99 [95%-20.397 - 8.411]	11.03 [95%CI -4.537-26.595]	*-17.02 -33.36 -0.69]
**IDAA1c**	**GABA**	**GABA/GAD**	**Placebo**	**GABA vrs Placebo**	**GABA vrs GABA/GAD**	**GABA/GAD vrs placebo**	**GABA vrs Placebo**	**GABA vrs GABA/GAD**	**GABA/GAD vrs placebo**
Baseline	13.30 [95%CI 12.36 - 14.23]	12.33 [95%CI 11.08 - 13.59]	13.31 [95%CI 12.24 - 14.38]	0.980	0.226	0.241	*-0.02 [95%CI -1.44 - 1.41]	0.96 [95%CI -0.60 - 2.5]	*-0.99 [95%CI -2.63 - 0.67]
1 month	9.06 [95%CI 8.60 - 9.53]	8.34 [95%CI 7.68 - 9.00]	8.81 [95%CI 8.25 - 9.36]	0.476	0.079	0.289	0.26 [95%CI -0.46 - 0.98]	0.72 [95% -0.09 - 1.53]	*-0.46 {95%CI -1.33 - 0.40]
5 months	9.20 [95%CI 8.70 - 9.71]	7.93 [95% 7.30 - 8.56]	8.16 [95%CI 7.61 - 8.72]	0.007	0.002	0.581	1.04 [95%CI 0.29 - 1.79]	*-1.27 [95%CI 0.46 -2.08]	*-0.23 [95%CI -1.08- 0.61]
8 months	9.75 [95%CI 9.10 - 10.40]	8.75 [95%CI 7.93 - 9.56]	8.76 [95%CI 7.00 - 9.53]	0.053	0.059	0.976	0.99 [95%CI -0.015 - 1.99]	1.01 [95%CI -0.038 - 2.05]	*-0.02 [95%CI -1.14 - 1.10]
12 months	10.35 [95%CI 9.70 - 11.001]	9.47 [95%CI 8.60 - 10.33]	9.17 [95%CI 8.437 - 9.91]	0.020	0.107	0.612	1.18 [95%CI 0.19 - 2.17]	0.89 [95%CI -0.20 - 1.97]	0.29 [95%CI -0.85 - 1.43]

Note:Statistical differences for primary and secondary outcomes. Results are given as mean ± 95% CI. Statistical differences were by two-tailed analysis of variance. Source data are provided as a Source Data file.

Supplementary Table 2: Effect of HLA risk haplotype on c-peptide response in treatment groups

parameter	Study visit (months)	study group	Presence of HLA-DR3-DQ2	Absence of HLA DR3 - DQ2	p value presence vrs absence HLA DR3 - DQ2	Presence of HLA-DR3-DQ2 (but no DR4-DQ8)	Absence of HLA-DR3-DQ2 (but no DR4-DQ8)	p value presence vrs absence HLA-DR3-DQ2 (but no DR4-DQ8)
**Fasting c-peptide**	Baseline	GABA	0.65±0.45 (0.06-1.65) n=19	0.88±0.64 (0.21-2.20) n=17	0.216	0.74±0.55 (0.06-1.65) n=9	0.77±0.56 (0.10-2.20) n=27	0.908
		GABA/GAD	0.76±0.50 (0.24-2.02) n= 12	0.73±0.44 (0.04-1.36) n=9	0.868	0.82±0.81 (0.26-2.02) n=4	0.73±0.38 (0.04-1.36) n=17	0.735
		Placebo	0.71±0.74 (0.10-3.15) n=16	0.77±0.43 (0.16-1.54) n=16	0.792	0.68±0.42 (0.28-1.30) n=5	0.75±0.65 (0.10-3.15) n=24	0.842
	5 months	GABA	0.58±0.42 (0.10-1.30) n=18	0.79±0.65 (0.04-2.20) n=16	0.266	0.63±0.49 (0.10-1.30) n=8	0.70±0.57 (0.04-2.20) n=26	0.742
		GABA/GAD	0.69±0.41 (0.20-1.63) n=12	0.86±0.43 (0.30-1.70) n=8	0.382	0.83±0.60 (0.20-1.63) n=4	0.73±0.38 (0.20-1.70) n=16	0.683
		Placebo	0.67±0.50 (0.04-1.65) n=16	0.64±0.68 (0.10-2.23) n=11	0.915	1.09±0.54 (0.38-1.65) n=5	0.56±0.53 (0.04-2.23) n=22	0.058
	12 months	GABA	0.42±0.50 (0.04-2.05) n=18	0.52±0.37 (0.04-1.17) n=14	0.542	0.51±0.68 (0.04-2.05) n=8	0.45±0.36 (0.04-1.17) n=24	0.752
		GABA/GAD	0.54±0.43 (0.10-1.40) n=12	0.51±0.36 (0.04-1.23) n=7	0.898	0.72±0.48 (0.26-1.23) n=4	0.47±0.34 (0.04-1.40) n=15	0.255
		Placebo	0.60±0.55 (0.04-1.40) n=14	0.53±0.65 (0.02-2.50) n=11	0.755	0.69±0.36 (0.35-1.05) n=4	0.53±0.63 (0.02-2.50) n=21	0.647
**AUC c-peptide**	Baseline	GABA	1.66±0.99 (0.20- 3.79) n=19	2.10±1.42 (0.59- 5.20) n=17	0.290	1.82±1.28 (0.20-3.79) n=9	1.89±1.21 (0.59-5.20) n=27	0.884
		GABA/GAD	2.03±1.06 (0.82- 4.38) n=12	2.17±1.39 (0.56- 5.24) n=9	0.796	2.08±1.61 (0.82-4.38) n=4	2.10±1.12 (0.56-5.24) n=17	0.983
		Placebo	1.93±1.50 (0.54- 6.56) n=16	1.87±1.06 (0.71- 4.21) n=13	0.916	2.35±0.89 (0.78-3.00) n=5	1.81±1.37 (0.54-6.56) n=24	0.412
	12 months	GABA	0.94±1.34 (0.04- 5.69) n=18	1.00±0.73 (0.04- 2.19) n=14	0.879	1.18±1.88 (0.08-5.69) n=8	0.90±0.73 (0.04-2.19) n=24	0.539
		GABA/GAD	1.09±0.80 (0.08- 2.96) n=12	1.13±0.95 (0.19- 3.04) n=7	0.936	1.31±0.67 (0.44-1.87) n=4	1.05±0.88 (0.08-3.04) n=15	0.589
		Placebo	1.19±1.60 (0.06- 6.09) n=14	1.71±1.70 (0.04- 4.06) n=11	0.441	1.43±1.13 (0.49-2.98) n=4	1.42±1.74 (0.04-6.09) n=21	0.996

Note:. Results are presented as mean±95%CI. Statistics were by two-way analysis of variance. Baseline = Time 0 prior to treatments. Source data are provided as a Source Data file

Supplementary References

1. Bachmanov, A.A., Reed, D.R., Beauchamp, G.K. & Tordoff, M.G. Food intake, water intake, and drinking spout side preference of 28 mouse strains. *Behav Genet* **32**, 435-443 (2002).
2. DEPARTMENT OF HEALTH AND HUMAN SERVICES Food and Drug Administration Draft Guidance for Industry and Reviewers on Estimating the Safe Starting Dose in Clinical Trials for Therapeutics in Adult Healthy Volunteers; Availability. 1008016u1005861 (COMTEX News Network, Inc., 2003).
3. Nair, A.B. & Jacob, S. A simple practice guide for dose conversion between animals and human. *J Basic Clin Pharm* **7**, 27-31 (2016).
4. Reagan-Shaw, S., Nihal, M. & Ahmad, N. Dose translation from animal to human studies revisited. *FASEB J* **22**, 659-661 (2008).
5. Li, J.*, et al.* Study of GABA in Healthy Volunteers: Pharmacokinetics and Pharmacodynamics. *Front Pharmacol* **6**, 260 (2015).
6. Ben-Othman, N.*, et al.* Long-Term GABA Administration Induces Alpha Cell-Mediated Beta-like Cell Neogenesis. *Cell* **168**, 73-85 e11 (2017).
7. Feng, A.L.*, et al.* Paracrine GABA and insulin regulate pancreatic alpha cell proliferation in a mouse model of type 1 diabetes. *Diabetologia* **60**, 1033-1042 (2017).
8. Tian, J.*, et al.* Gamma-aminobutyric acid inhibits T cell autoimmunity and the development of inflammatory responses in a mouse type 1 diabetes model. *J Immunol* **173**, 5298-5304 (2004).
9. Hwang, I.*, et al.* GABA-stimulated adipose-derived stem cells suppress subcutaneous adipose inflammation in obesity. *Proc Natl Acad Sci U S A* **116**, 11936-11945 (2019).
10. Tian, J.*, et al.* Oral treatment with gamma-aminobutyric acid improves glucose tolerance and insulin sensitivity by inhibiting inflammation in high fat diet-fed mice. *PLoS One* **6**, e25338 (2011).
11. Sohrabipour, S., Sharifi, M.R., Talebi, A., Sharifi, M. & Soltani, N. GABA dramatically improves glucose tolerance in streptozotocin-induced diabetic rats fed with high-fat diet. *Eur J Pharmacol* **826**, 75-84 (2018).
12. Prud'homme, G.J., Glinka, Y., Kurt, M., Liu, W. & Wang, Q. The anti-aging protein Klotho is induced by GABA therapy and exerts protective and stimulatory effects on pancreatic beta cells. *Biochem Biophys Res Commun* **493**, 1542-1547 (2017).
13. Untereiner, A.*, et al.* GABA promotes β-cell proliferation, but does not overcome impaired glucose homeostasis associated with diet-induced obesity. *Faseb j* **33**, 3968-3984 (2019).
14. Liu, W.*, et al.* Combined Oral Administration of GABA and DPP-4 Inhibitor Prevents Beta Cell Damage and Promotes Beta Cell Regeneration in Mice. *Front Pharmacol* **8**, 362 (2017).
15. Purwana, I.*, et al.* GABA promotes human beta-cell proliferation and modulates glucose homeostasis. *Diabetes* **63**, 4197-4205 (2014).
16. Mortensen, H.B.*, et al.* New definition for the partial remission period in children and adolescents with type 1 diabetes. *Diabetes Care* **32**, 1384-1390 (2009).
