# Supplementary material for: A randomized trial of oral gamma aminobutyric acid (GABA) or the combination of GABA with glutamic acid decarboxylase (GAD) on pancreatic islet endocrine function in children with newly diagnosed type 1 diabetes
Source: Nat Commun. 2022 Dec 24;13:7928. doi: 10.1038/s41467-022-35544-3 (PMC9790014; doi:10.1038/s41467-022-35544-3)
Supplement: Supplementary file 2 — Reporting Summary [file 41467_2022_35544_MOESM2_ESM.pdf]

nature portfolio

Corresponding author(s): Kenneth McCormick

Last updated by author(s): Oct 7, 2022

Reporting Summary

Nature Portfolio wishes to improve the reproducibility of the work that we publish. This form provides structure for consistency and transparency in reporting. For further information on Nature Portfolio policies, see our Editorial Policies and the Editorial Policy Checklist.

Statistics

For all statistical analyses, confirm that the following items are present in the figure legend, table legend, main text, or Methods section.

n/a	Confirmed	
☐	☒	The exact sample size (n) for each experimental group/condition, given as a discrete number and unit of measurement
☒	☐	A statement on whether measurements were taken from distinct samples or whether the same sample was measured repeatedly
☐	☒	The statistical test(s) used AND whether they are one- or two-sided<br>*Only common tests should be described solely by name; describe more complex techniques in the Methods section.*
☐	☒	A description of all covariates tested
☒	☐	A description of any assumptions or corrections, such as tests of normality and adjustment for multiple comparisons
☐	☒	A full description of the statistical parameters including central tendency (e.g. means) or other basic estimates (e.g. regression coefficient) AND variation (e.g. standard deviation) or associated estimates of uncertainty (e.g. confidence intervals)
☐	☒	For null hypothesis testing, the test statistic (e.g. F, t, r) with confidence intervals, effect sizes, degrees of freedom and P value noted<br>*Give P values as exact values whenever suitable.*
☒	☐	For Bayesian analysis, information on the choice of priors and Markov chain Monte Carlo settings
☒	☐	For hierarchical and complex designs, identification of the appropriate level for tests and full reporting of outcomes
☐	☒	Estimates of effect sizes (e.g. Cohen's d, Pearson's r), indicating how they were calculated

Our web collection on statistics for biologists contains articles on many of the points above.

Software and code

Policy information about availability of computer code

Data collection	This study utilized REDCap (Research Electronic Data Capture, version 12.3.3 https://www.project-redcap.org), a software toolset and workflow methodology for electronic collection and management of clinical and research data, to collect and store data. The University of Alabama Birmingham Heersink School of Medicine and Information Technology Electronic Data Capture (https://www.uab.edu/medicine/dom/information-technology/services/redcap) was used as the central location for data processing and management.
Data analysis	Data analysis of for primary, secondary and post hoc outcomes as with SAS/STAT software, version 9.4 of the SAS System. Copyright, SAS Institute Inc. SAS and all other SAS Institute Inc. product or service names are registered trademarks or trademarks of SAS Institute Inc., Cary, NC, USA.<br>Graphs were prepared with GraphPad Prism 9.0 for Windows, GraphPad Software, San Diego, CA, USA, www.graphpad.com.<br>Spearman correlations and Fisher's exact analyses or as noted were by GraphPad.

For manuscripts utilizing custom algorithms or software that are central to the research but not yet described in published literature, software must be made available to editors and reviewers. We strongly encourage code deposition in a community repository (e.g. GitHub). See the Nature Portfolio guidelines for submitting code & software for further information.

Data

Policy information about availability of data

All manuscripts must include a data availability statement. This statement should provide the following information, where applicable:

- Accession codes, unique identifiers, or web links for publicly available datasets
- A description of any restrictions on data availability
- For clinical datasets or third party data, please ensure that the statement adheres to our policy

The clinical data that support the findings in this study are available on reasonable request. Source data are published along side the paper.

Field-specific reporting

Please select the one below that is the best fit for your research. If you are not sure, read the appropriate sections before making your selection.

☒ Life sciences ☐ Behavioural & social sciences ☐ Ecological, evolutionary & environmental sciences

For a reference copy of the document with all sections, see nature.com/documents/nr-reporting-summary-flat.pdf

Life sciences study design

All studies must disclose on these points even when the disclosure is negative.

Sample size	The sample size for the proposed study was 110 children; 30 in the treatment group of active GABA and active GAD-alum, 43 in the treatment group receiving active GABA and placebo GAD-alum, and 37 in the placebo group. For the primary comparison of the 12-month post-baseline C-peptide measurements between these groups, assuming an α of 0.05 and a mean (SD) C-peptide AUC of 1.0 (0.4) this sample size yields a ~97% power to detect a 50% difference.
Data exclusions	Adhering to nearly all new-onset Type 1 diabetes study protocols, participants with baseline serum c-peptide values <0.6 ng/ml, both fasting and mixed meal stimulated, were excluded prior to analysis. Patients were randomized before exclusion because serum was batched and frozen at -80°C until assay at study completion.
Replication	Sample volumes were sometimes limited because this was an outpatient pediatric study (ages 4-17 years) wherein the amount of blood that could be reasonably obtained by veno-puncture was challenging, particularly in the youngest participants. As a result, replicates were not routine. Blood analyses were measured either in our university core endocrine/metabolic lab (hormones), hospital lab (HbA1c and glucose) or by a reliable and accredited commercial endocrine lab (antibody assays) using the most efficient pediatric sample volumes possible.
Randomization	A total of 97patients including, 25 (GABA + GAD-alum group), 41 (GABA group) and 31 (placebo group), were enrolled. The study was double-blinded with randomization under the stewardship of the Children's Hospital research pharmacist. Subjects were randomized in a 1:1:1 ratio (GABA:GABA/GAD:Placebo) for the first 75 patients and in a 2:1 ratio (GABA:placebo) for the final 22patients. This second protocol was a consequence of unanticipated additional funding that afforded trial extension for the GABA versus placebo groups only. Only the un-blinded pharmacist knew the pre-set randomization list (generated by using a computerized procedure).
Blinding	This was a double-blind, placebo-controlled clinical trial

Reporting for specific materials, systems and methods

We require information from authors about some types of materials, experimental systems and methods used in many studies. Here, indicate whether each material, system or method listed is relevant to your study. If you are not sure if a list item applies to your research, read the appropriate section before selecting a response.

Materials & experimental systems

n/a	Involved in the study	
☒	☐	Antibodies
☒	☐	Eukaryotic cell lines
☒	☐	Palaeontology and archaeology
☒	☐	Animals and other organisms
☐	☒	Human research participants
☐	☒	Clinical data
☒	☐	Dual use research of concern

Methods

n/a	Involved in the study	
☒	☐	ChIP-seq
☒	☐	Flow cytometry
☒	☐	MRI-based neuroimaging

Human research participants

Policy information about studies involving human research participants

Population characteristics	All participants had type 1 diabetes (T1D) diagnosed within six weeks prior to enrollment. The age range was 4-18 years of age and the percent male patients was 54%. Ethnicity was 90% Caucasian, 7% African American, 2% Hispanic and 1% Native American.
Recruitment	Recruitment strategies involve broad outreach through emails, Internet material, posters and local presentations. All families of children with newly-diagnosed T1DM at our university children's hospital received an information packet of general resources relevant to the diagnosis, including our study information document. Formal recruitment discussions were timed for late afternoon or evening, a day or so after admission. This was a more agreeable interval for families and children as the initial trepidation of diagnosis had diminished and the children had recovered from their presenting symptoms. Most often a physician investigator (KM, AM, or CHM) explained the study and answered questions. All children with new-onset diabetes were admitted to the hospital for insulin initiation, therefore, all received the same introductory study information so as to avoid potential selection bias. At discharge families were invited to contact the research coordinator for further information. Out of state participants contacted the research coordinator via clinicaltrials.gov and included 11 patients from 8 states other than Alabama (AZ, GA, MS, MO, NC, ND, TX, VA).
Ethics oversight	The project was approved by the University of Alabama Birmingham Institutional Review Board(IRB). The trial was monitored threefold by: 1) a university-based independent Data Safety Monitoring Board (DSMB), 2) the UAB Center for Clinical and Translational Science (CCTS) monitor, and, 3) by a sponsor-appointed trial monitor from QA Partners, LLC. Written informed consent was obtained from each participant or from the participant's parent or legal guardian. Also, each participant assented. Participants received a $60 gift card as compensation for every blood draw.

Note that full information on the approval of the study protocol must also be provided in the manuscript.

Clinical data

Policy information about clinical studies

All manuscripts should comply with the ICMJE guidelines for publication of clinical research and a completed CONSORT checklist must be included with all submissions.

Clinical trial registration	Clinicaltrials.gov, NCT02002130
Study protocol	The full protocol is available on clinicaltrials.gov.
Data collection	Data was collected and promptly entered into a secure, university-based REDcap database between 02March2015 (first patient enrolled) and 12 October 2021. The last patient was enrolled in July 2018. The last participant completed the study on 24June2019.
Outcomes	Primary Outcome Measures:<br>Compare the effect of oral GABA or oral GABA/GAD combination administration on pancreatic beta cell function by quantitative C-peptide secretion [Time Frame: 12 months after baseline]<br>This will be assessed by meal stimulated c-peptide secretion in treatment cohorts compared to age matched placebo controls before and after 1 year of treatment.<br><br>Secondary Outcome Measures:<br>1. Compare the effect of oral GABA or GABA/GAD administration on fasting and meal stimulated glucagon and pro-insulin levels. [Time Frame: 12 months after baseline]<br>This will be assessed by meal stimulated glucagon and pro-insulin secretion in treatment cohorts compared with age matched placebo controls before and after 1 year of treatment.<br>2. Compare the effect of oral GABA or GABA/GAD administration on total daily insulin usage by participants and corrected Hemoglobin A1C. [Time Frame: 12 months after baseline]<br>This will be assessed by measuring insulin usage and corrected Hemoglobin A1C linearly for each participant, and calculating insulin dose adjusted A1c (IDAA1c) in addition to comparison with age matched controls.<br>3. Compare the effect of oral GABA or oral GABA/GAD administration on indices of immune system function. [Time Frame: 12 months after baseline]<br>This will be assessed by measuring immunologic markers of inflammation in peripheral blood mononuclear cells over the 12 month treatment course for each patient and comparing to age matched placebo controls.<br>4. Compare the effect of oral GABA or oral GABA/GAD administration on diabetes related autoantibodies [Time Frame: 12 months after baseline]<br>This will be assessed by measuring GAD65, ICA512, and Zinc Transporter 8 antibodies throughout the treatment period.<br><br>Exploratory Outcomes<br>1. Effect of diabetes HLA-risk subtype on the primary outcome, fasting and meal-stimulated c-peptide, in the treatment groups.<br>2. Fasting and post meal-stimulated serum GABA levels in the treatment groups.<br>3. Fasting and post meal-stimulated proinsulin/c-peptide ratio in the treatment groups
